# 

**Published:** 1859-07

**Authors:** 


### RECEIPTS FOR THE JOURNAL, UP TO JULY 4, 1859.

### ILLINOIS.

| | | | |
|---|---|---|---|
| Dr. D. D. Wait, | vol. 2, 1859, | | $1 00 |
| " Thos. D. Washburn, | " | " | 2 00 |
| " B. F. Haller, | " | " | 2 00 |
| " —— Folke, | " | " | 2 00 |
| " A. Taylor, | " | " | 2 00 |
| " A. L. Kimber, | " | " | 2 00 |
| " W. R. Fox, | " | " | 2 00 |
| " John Crim, | " | " | 2 00 |
| " A. Rodenstad, | " | " | 2 00 |
| " W. A. Looney, | " | " | 2 00 |
| " J. S. Bullock, | vol. 1 & 2, 1858-9, | | 3 00 |

### INDIANA.

| | | | |
|---|---|---|---|
| Dr. A. Heavenridge, | vol. 2, 1859, | | $2 00 |
| " Dudley Rogers, | " | " | 2 00 |

### IOWA.

| | | | |
|---|---|---|---|
| Dr. S. L. Hazen, | vol. 2, 1859, | | $2 00 |
| " Myron Underwood, | " | " | 2 00 |
| " D. G. Henlan, | " | " | 1 00 |

### WISCONSIN.

| | | | |
|---|---|---|---|
| Drs. Cask and Rice, | vol. 2, 1859, | | $2 00 |
| Dr. R. W. Earl, | " | " | 2 00 |
| " James Cody, | " | " | 2 00 |
| " C. J. Taggart, | " | " | 2 00 |

